# Presumptive Haematomyelia Secondary to Warfarin Toxicosis in a Dog

**DOI:** 10.1155/2022/8349085

**Published:** 2022-08-04

**Authors:** Carlos Blanco, Meritxell Moral, Juan José Minguez, Valentina Lorenzo

**Affiliations:** ^1^Neurología Veterinaria, Getafe, 28906 Madrid, Spain; ^2^Scarsdale Vets-Pride Veterinary Centre, Derby DE24 8HX, UK

## Abstract

A 3-year-old male entire Boxer was presented for a 6-day history of progressive symmetric nonambulatory tetraparesis with diffuse spinal hyperesthesia. Eight days prior to admission, the dog ingested warfarin accidentally, exhibiting systemic clinical signs of intoxication 2 days later. Upon referral, the dog was nonambulatory with paretic thoracic limbs and plegia with absent nociception on pelvic limbs, spinal reflexes were decreased to absent in all four limbs, and urinary and faecal incontinence were noticed. Magnetic resonance imaging (MRI) of the cervical, thoracic, and lumbar regions of the vertebral column revealed intramedullary lesions extending from the first cervical segments to the conus medullaris consistent with extensive intramedullary haemorrhages. Despite management with vitamin K1 and physiotherapy, 6 weeks later, improvement was limited to thoracic limb motor function, and euthanasia was elected. This case reports an extensive presumptive haematomyelia with severe neurological deficits suspected to be secondary to warfarin intoxication in a dog.

## 1. Introduction

Intramedullary haemorrhage (haematomyelia) is rarely reported in veterinary medicine [1], with most cases secondary to spinal cord trauma [2, 3]. Nontraumatic causes have been described as a result of vascular malformations [3], spinal tumors [4], parasitic diseases such as *Angiostrongylus vasorum* [5] and *Leishmania infantum* [6], juvenile polyarteritis syndrome [7], and congenital coagulopathies such as Von Willebrand disease and hemophilia A and B [8, 9]. This case reports an extensive presumptive haematomyelia with severe neurological deficits suspected to be secondary to warfarin intoxication in a dog.

## 2. Case Presentation

A 3-year-old male entire Boxer was referred for spinal pain and symmetric nonambulatory tetraparesis progressing for six days. Eight days before consultation, the dog inadvertently had ingested a rodenticide that was kept by the owner in the garage. Two days after the rodenticide ingestion, the dog was hospitalized because of generalized hyperesthesia, hematuria, hematochezia, tachypnoea, and pelvic limb weakness. On thoracic radiographs, an alveolar pattern with patchy distribution was present, consistent with pulmonary infiltration, and this was suspected to represent haemorrhage. Blood analysis performed on the day of hospitalization showed moderated thrombocytopenia (103 K/mcL, reference range 148-484) and altered coagulation profile (PT 40.9 sec, reference range 14-20; APTT 140.7 sec, reference range 94-123). Immediate treatment consisted of oxygen support, analgesia (buprenorphine 0.01 mg/kg IM q8h), and vitamin K1 (5 mg/kg, initially intramuscularly, and then 2.5 mg/kg SC q12h). The patient remained hospitalized for six days, and at that time, it was referred to our center. On presentation, the dog was unable to control the excretion of urine or faeces. Tachycardia, dyspnoea, diffuse spinal hyperesthesia, and traces of hematochezia in the perineal region were noticed on physical examination.

On neurological examination, the dog was in lateral recumbency with flaccid muscle tone, the thoracic limbs were paretic, and the pelvic limbs were plegic with absent nociception. Spinal reflexes were decreased to absent in all four limbs, and perineal reflex was absent. The history and clinical findings suggested the presence of a multifocal spinal cord lesion affecting at least the two (C6-T2 and L4-S3) intumescences. Vascular myelopathy (mainly haemorrhage) was considered most likely. Inflammatory myelopathies, ascending/descending myelopathy secondary to disc herniation or neoplasia with secondary bleeding, were also considered.

Biochemistry revealed mild hypoalbuminemia (2 g/dl, reference range 2.5-4), and coagulation profile was within normal limits (PT 14.2 sec, reference range 14-20; APTT 112.5 sec, reference range 94-123).

**Figure 1:**
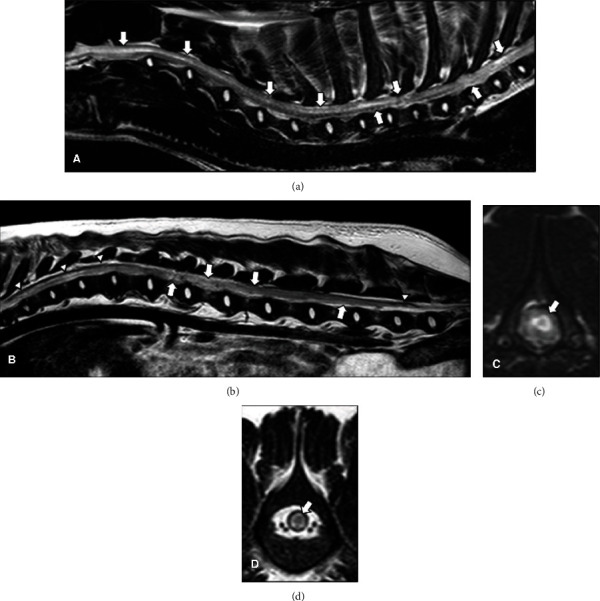
MRI T2-weighted sagittal planes (a, b) of the cervical, thoracic, and lumbar vertebral column showing an extensive heterogeneous intramedullary lesion (arrows) and the presence of a hypointense dorsal rim suggesting subdural haematoma (small arrows on (b)). On the transverse planes at the level of C2 (c) and L5 (d), there are similar isointense lesions surrounded by a hyperintense rim (arrows). These signal patterns are consistent with a haematomyelia changing from early subacute to late subacute with intra- and extracellular methemoglobin.

**Figure 2:**
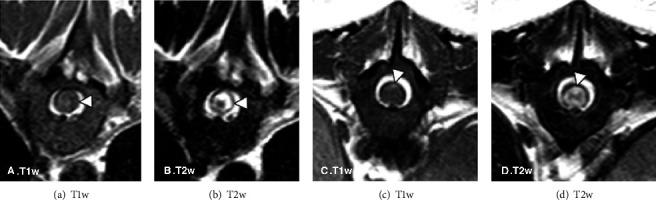
MRI transverse planes at T8 level (a, b). The images show an intramedullary lesion which is hyper- to isointense with and hypointense rim on T1-weighted images (a) and hypointense with a hyperintense rim on T2-weighted images (b). This pattern is suggestive of early subacute haematoma surrounded by fluid. At T12 level (c, d), the images show an intramedullary lesion which is mildly hyperintense on T1-weighted images (c) and hyperintense on T2 (d). These findings are suggestive of late subacute haematoma.

**Figure 3:**
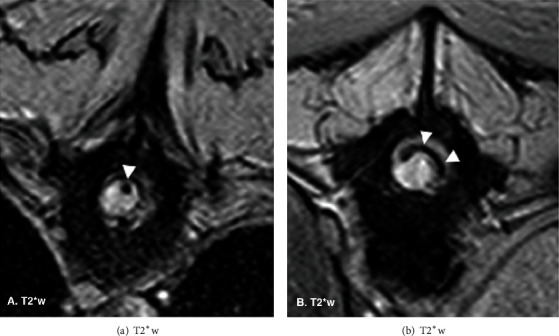
MRI gradient eco transverse planes at T9 level show an intramedullary round focal signal void in the dorsal funiculus (arrow in (a)) characteristic of haemorrhage and a dorsolateral hypointense rim at T12 level (arrows in (b)) compatible with subdural haematoma, with hemosiderin creating the magnetic susceptibility variation depicted on this sequence.

The vertebral column including cervical, thoracic, and lumbar areas was evaluated by MRI (Gyroscan Intera 1,5 T, Philips, Eindhoven, The Netherlands). Sagittal and transverse T2-weighted, T1-weighted, and gradient echo sequences were acquired. Intraparenchymal heterogeneous spinal cord lesions were found along the entire spinal cord from first cervical segments to the *conus medullaris* (Figures 1(a)–1(d)). The lesions were mainly isointense or slightly hyperintense compared to normal gray matter in T1-weighted images (Figures 2(a)–2(c)), hyperintense in T2-weighted images (Figures 2(b) and 2(d)), and hypointense (signal void) in T2^∗^-weighted images (Figure 3(a)) consistent with early to late subacute haemorrhages. In addition, a hypointense line was identified on T2 and T2^∗^-weighted images in the dorsolateral subdural space along the caudal thoracic and lumbar vertebral column, characteristic of subdural haematoma.

Based on the recent history of warfarin ingestion, the clinical data, and MRI findings, a diagnosis of extensive spinal cord haemorrhage secondary to warfarin coagulopathy was postulated. Other potential underlying causes for intramedullary haematomyelia such as disc herniation, neoplasia, and vascular malformation were excluded based on the MRI results. The dog was hospitalized and remained on treatment with vitamin K1 (2.5 mg/kg BID SC) and buprenorphine (0.01 mg/Kg IM q8h) for three days alongside implementation of physiotherapy. At the end of this hospitalization, the dog was found to be neurologically stable with no evidence of pain. Oral treatment with vitamin K1 (2.5 mg/kg/12 h) was prescribed for another week together with professional physiotherapy, and the patient was discharged home for ongoing care. A progressive improvement in thoracic limb motor function was observed during the first two weeks after discharge. Unfortunately, no improvement was noticed on pelvic limbs' motor function, or nociception, nor in faecal and urinary incontinence for the following 6 weeks. Euthanasia was ultimately elected by the owners owing to concern for the dog's quality of life. Inability to obtain permission from the owners to perform a postmortem examination meant pathological studies were not performed.

## 3. Discussion

Haemorrhage in the central nervous system by anticoagulant therapy in human medicine is a rare and potentially life-threatening condition with intraspinal haemorrhage being much less common than intracerebral or intracranial subdural haemorrage. Depending on its location, intraspinal haemorrhage can be epidural, subdural, subarachnoid ,or intramedullary, with intramedullary haemorrhage (haematomyelia) being the rarest form [10]. Haematomyelia, and in particular nontraumatic haematomyelia, is rarely reported in veterinary medicine [1–3]. Previously reported underlying causes for nontraumatic haematomyelia include vascular malformations [3], spinal tumors [4], parasites such as *Angiostrongylus vasorum* [5] and *Leishmania infantum* [6], and also related to juvenile polyarteritis syndrome [7] and congenital coagulopathies such as Von Willebrand disease or hemophilia A and B [8, 9]. Recently, a case of suspected primary haematomyelia has also been described in a French Bulldog with no identified underlying cause [1]. In human medicine, haematomyelia of spontaneous nontraumatic nature is also described as an uncommon condition mainly associated with hemophilia and in patients on anticoagulant treatment [10–12]. The use of warfarin in human medicine as an anticoagulant is associated with an increased risk of spinal subdural haematomas. Compared with other anticoagulants, warfarin is not only associated with a higher risk of intracranial subdural haematoma but also a worse prognosis by comparison to cases of intracranial haemorrhage due to another cause [13, 14]. Warfarin competitively inhibits the vitamin K epoxide reductase complex 1 (VKORC1), which is essential for activating vitamin K. Vitamin K is an essential cofactor for the hepatic synthesis of multiple clotting factors including II, VII, IX, and X and factors protein C and protein S [15]. Only active vitamin K can perform carboxylation, which is the process of turning “spent” coagulation factors into active ones [16, 17]. As such, the main adverse effect of warfarin is haemorrhage, which is not predictable based on the dosage administered. Anticoagulant rodenticides initially cause prolongation of the prothrombin time (PT), at 24-36 hours postingestion. This coincides with a depletion of Factor VII which has the shortest half-life of all the vitamin K-dependent factors (6.2 h in the dog) and whose activity is measured by the PT [16]. Prolongation of the aPTT follows when there is depletion of other coagulation factors [17, 18]. In the case reported above, the normal coagulation times recorded at the second blood test are consistent with the findings of previous studies where all dogs recovered physiological ranges at 4-5 days after administration of vitamin K [16, 17].

As in the case described here, hyperesthesia together with neurological deficits has also been reported in all the previous cases of haematomyelia described in veterinary medicine with hemophilia A and B, in two of the four cases with *Angiostrongylus Vasorum,* in a case of juvenile polyarteritis syndrome, and other cases of unknown origin [5, 7–9]. Haematomyelia has been described in human medicine as an abrupt spinal syndrome, with intense local or radicular pain related to motor and sensory deficits, which in time and depending on its location [10] could be accompanied by urinary and/or faecal sphincter atony.

Some of the possible mechanisms of hyperesthesia in intramedullary lesions include neurochemical and inflammatory changes as well as mechanical changes, which affect the dorsal horn on the spinal cord [19]. Neurochemical and inflammatory changes due to altered neurotransmitter modulation could occur due to the remodeling of the dorsal horn [20, 21]. Alternatively, mechanical alterations due to bleeding may induce a mass effect with acute CSF obstruction, which can also contribute to stretching of the meninges, dorsal nerve roots, or both, leading to discomfort or pain [19]. Finally, spinal subdural haematomas can cause neurological signs and pain secondary to meningeal irritation and compression of the cord or nerve roots [11–13].

Interestingly, pain was not reported in a case of suspected primary haematomyelia [1], and moreover, in an article of three dogs with haematomyelia of unknown origin, two of them did not showed pain [2]. Similarly, in human medicine, a patient treated with anticoagulants developed an epidural haematoma without signs of pain [22]. Only in this last report it is postulated that factors such as immobilization after surgery and epidural analgesia may mask the clinical signs and delay diagnosis.

With regard to the biochemical abnormalities detected, we believe that protein loss secondary to the reported hematochezia represents the most likely cause for hypoalbuminemia in this case.

Among the diagnostic imaging modalities available in veterinary medicine, MRI is the most reliable imaging modality for detecting spinal haemorrage and also holds the potential of determining the source of bleeding as it is the best way to pinpoint associated spinal lesions [1–3, 11, 23, 24]. Acute haemorrhage is characterized by the presence of deoxyhemoglobin, depicted on high field MRI as discrete areas of hypointensity in T2-weighted and gradient echo T2^∗^ sequences and isointensity on T1W [23, 24]. Methemoglobin begins to form three days after the initial haemorrhage, but this conversion from deoxyhemoglobin to methemoglobin can be delayed in the hypoxic tissue of the injured spinal cord [24–28]. In the subacute-early stage (3-7 days), the methaemoglobin is intracellular, the signal on T1-weighted images changes from isointense to hyperintense, and the lesions remain hypointense on T2w (Figures 2(a) and 2(b)). In the subacute-late phase (1-2 weeks), the methaemoglobin is mainly extracellular, and the lesions become hyperintense in both T1w and T2w [23] (Figures 2(c) and 2(d)). In canine spinal cord injury, the methaemoglobin pattern is most commonly recognised as cases are not usually identified and thus imaged at the stage of acute haemorrhage [24]. Variable MRI patterns in haemorrhage are not only related to hemoglobin degradation but also to the presence of gliosis, oedema [23] (Figures 2(a) and 2(b)), or the presence of a syrinx adjacent to the lesion which can cause an abnormal signal in the cord parenchyma [25]. Iron plays a major role in these imaging findings as it is present in a high concentration in haemorrhage, and its magnetic properties vary according to its biochemical form, oxidative state, and spatial distribution. Computed tomography (CT) imaging findings can also be suggestive of intramedullary haemorrhage. In early subacute to late subacute stages, the imaging findings change from a hyperattenuating lesion with a hypoattenuating ring of oedema to an isoattenuating lesion; however, this imaging modality is considered less likely to provide indications of the underlying cause such as neoplasia, inflammation, or vascular abnormalities [9].

In the present case, the MRI study was performed at eight days after warfarin poisoning, by which time the lesions extended over the entire length of the spinal cord showing different degrees of haemoglobin oxidation, from early subacute (3 days to 1 week) to late subacute (1 week to 2 weeks). The imaging features of spinal haematoma are depicted in T1w, T2w, and gradient echo imaging sequences [23]. In the present case, the features of the lesion on these sequences together with the history were compatible with haematomyelia; therefore, it was considered that postcontrast images were unlikely to provide additional diagnostic information, in addition to which, given the general condition of the dog, the increased anaesthetic time required to obtain them could not be justified.

The vast majority of CNS haemorrhages reported in humans are located intracranially, with only 10% within the vertebral canal and/or spinal cord. The most frequent location in children is C1-C5 spinal segments unlike adults, in which the most frequently affected location is the last cervical and thoracolumbar segments, which are associated with high mortality [29, 30]. These patients can show rapid progressive deterioration, which highlights the importance of timely diagnosis and treatment.

Treatment may include surgical removal of the haematoma, but this is dependent the localisation and extent of the lesion [3, 11, 12]. In the current case, surgery was not considered as the haemorrhagic lesions were intraparenchymal and affected most of the spinal cord segments.

Medical treatment of warfarin poisoning is based on vitamin K1 administration with the aim of allowing ongoing carboxylation of vitamin K-dependent factors. Intramuscular/subcutaneous injections of vitamin K1 at doses of 5 mg/kg should be administered as a matter of urgency in order to achieve high levels of vitamin K in the liver as soon as possible. Vitamin K will diffuse to the sites of clotting factor production when present in concentrations 40 times greater than normal [31]. It is recommended that the vitamin K injections are subsequently followed by the oral administration of 2.5 mg/kg/q12h for seven days [32]. A recent study with 4 dogs describes intravenous administration of vitamin K to rapidly reverse the clotting factor deficiency; however, this may carry a substantial risk of anaphylactoid reaction [33].

In the current case, the administration of vitamin K1 was started 48 hours after the poisoning and is considered the likely cause for normalization of the clotting times by the point of referral. In spite of this, vitamin K administration did not avoid the progression of the neurological signs, likely because these were due to spinal haemorrhages which had already occurred. Another reported option for rapid provision of coagulation factors in these cases is through transfusion of a blood product containing plasma [34]. A blood transfusion was not performed in this case given that the administration of vitamin K1 IV has been shown to facilitate the restoration of coagulation factors in less time [33], which in turn has the potential to reduce the degree of haemorrhage and thus the subsequent neurological deficits.

Immediately after diagnosis, the dog was treated with intense physiotherapy and recovered motor function of the thoracic limbs; however, paraplegia alongside urinary and faecal incontinence remained. This is in line with previous reports where despite improvement of motor function, urinary and/or faecal incontinence persisted even with a focal lesion [2].

In humans, treatment of epidural or subdural haematomas caused by warfarin overdose may be conservative or surgical. The conservative option is based on discontinuing warfarin treatment and restoring coagulation using human prothrombin complex concentrate [35] (proven to be safe and effective in patients with intracranial haematomas) and vitamin K1 [36]. Decompressive surgery may be performed in the face of a spinal subdural haematoma if deficits prevail or progress, with a high recovery rate if performed within the first 72 hours [11]. Physiotherapy has proved to be an effective method of improving motor function in human patients with intramedullary haemorrhage with or without surgery [35] but in most cases, some disabilities persist [37, 38].

The association between the extent of spinal haemorrhage and the severity of the signs have not been evaluated in veterinary medicine [24]; however, we would postulate that rapid intervention would likely limit the haemorrhage extension therefore reducing the severity of the neurological deficits and improving outcome.

Intramedullary nontraumatic spinal haemorrhage is extremely rare but may have devastating consequences, and warfarin poisoning can be an important risk factor for this condition. This case report describes an extensive presumptive haematomyelia with severe neurological signs after warfarin intoxication, and to the authors' knowledge, it serves as the first description of this condition in a dog. The main limitation in this case is the lack of postmortem examination.

## Data Availability

The data used to support the findings of this study were included in the article.
